# Antibiotic Resistance in *Salmonella* Typhimurium Isolates Recovered From the Food Chain Through National Antimicrobial Resistance Monitoring System Between 1996 and 2016

**DOI:** 10.3389/fmicb.2019.00985

**Published:** 2019-05-07

**Authors:** Xuchu Wang, Silpak Biswas, Narayan Paudyal, Hang Pan, Xiaoliang Li, Weihuan Fang, Min Yue

**Affiliations:** ^1^Hangzhou Center for Disease Control and Prevention, Hangzhou, China; ^2^CATG Microbiology and Food Safety Laboratory, Institute of Preventive Veterinary Medicine, College of Animal Sciences, Zhejiang University, Hangzhou, China; ^3^Zhejiang Provincial Key Laboratory of Preventive Veterinary Medicine, Hangzhou, China

**Keywords:** *Salmonella* Typhimurium, foodborne pathogen, antibiotic resistance, minimum inhibitory concentration, population diversity, fluoroquinolones

## Abstract

*Salmonella* is a major foodborne pathogen which causes widespread contamination and infection worldwide. *Salmonella* Typhimurium is one of the leading serovars responsible for human and animal salmonellosis, globally. The increasing rate of antibiotic resistance in *Salmonella* Typhimurium poses a significant global concern, and an improved understanding of the distribution of antibiotic resistance patterns in *Salmonella* Typhimurium is essential for choosing the suitable antibiotic for the treatment of infections. To evaluate the roles of animal and human in antibiotic resistance dissemination, this study aims to categorize 11,447 *S*. Typhimurium strains obtained across the food-chain, including food animals, retail meats and humans for 21 years in the United States by analyzing minimum inhibitory concentrations (MICs) values for 27 antibiotics. Random Forest Algorithm and Hierarchical Clustering statistics were used to group the strains according to their minimum inhibitory concentration values. Classification and Regression Tree analysis was used to identify the best classifier for human- and animal-populations’ isolates. We found the persistent population or multi-drug resistant strains of *S*. Typhimurium across the four time periods (1996∼2000, 2001∼2005, 2006∼2010, 2011∼2016). Importantly, we also detected that there was more diversity in the MIC patterns among *S*. Typhimurium strains isolated between 2011 and 2016, which suggests significant emergence of diversified multi-drug resistant strains. The most frequently observed (43%) antibiotic resistance patterns found in *S*. Typhimurium were tetra-resistant pattern ASSuT (ampicillin, streptomycin, sulfonamides, and tetracycline) and the penta-resistant pattern ACSSuT (ampicillin, chloramphenicol, streptomycin, sulfonamides, and tetracycline). Animals (mainly swine and bovine) are the major source for these two frequently found antibiotic resistance patterns. The occurrence of antibiotic resistant strains from humans and chicken is alarming. Strains were mostly susceptible to fluoroquinolones. Together, this study helped in understanding the expansion of dynamics of antibiotic resistance of *S*. Typhimurium and recommended fluoroquinolones as a possible treatment options against *S*. Typhimurium infection.

## Introduction

The burden of foodborne illnesses is tremendous, affecting 10% of global population with 33 million deaths annually (Havelaar et al., 2015). Numerous factors contribute to diarrheal diseases, and *Salmonella enterica* causes foodborne illnesses with significant public health impact. *Salmonella enterica* has a group of ∼2600 closely related bacteria defined by their mosaic combination of surface O and H antigens, so-called serovar. Based on the different pathogenic behaviors there are two groups of *Salmonella*, these are, typhoid *Salmonella* and non-typhoidal *Salmonella*. While typhoidal *Salmonella* can result in systemic infections with high fatal capabilities, non-typhoidal *Salmonella* infections are generally self-limiting (Su and Chiu, 2007; Gal-Mor et al., 2014). The emergence of pathogenic *Salmonella enterica* serovar Typhimurium (*S.* Typhimurium), armed with multiple antibiotic resistance (AR) in particular, presents a considerable threat to public health and food safety.

*Salmonella* Typhimurium, with its broader range of host tropism, is one of the top two serovars responsible for causing infections in human and animal worldwide (Hohmann, 2001; Herikstad et al., 2002; Foley and Lynne, 2008; Lan et al., 2009; Majowicz et al., 2010; Hendriksen et al., 2011). Throughout the last few decades, *S*. Typhimurium DT104 (DT104) evolved and disseminated rapidly across the globe (Helms et al., 2005; Lan et al., 2009). Genomic island (SGI-1) was suggested to be an important factor for the multi-drug resistance (MDR) phenotype in DT104 (Paul et al., 2016) and the acquisition of different resistance genes changed the bacterial fitness and virulence, which lead to emergence of highly virulent ST34 or ST313 clones (Okoro et al., 2012; Mather et al., 2018). The bacterial strains resistant to more than two antimicrobial drug classes are defined as multi-drug resistant. Importantly, DT104 isolates of animal were suggested to be different from the isolates of human population by comparative population genomic analysis. Additionally, the antibiotic resistance pattern between human and food–animal isolates population has some overlaps.

*Salmonella* Typhimurium is usually isolated from human, retail meats, and animal origins (Zhang et al., 2003; Kingsley et al., 2009; Izumiya et al., 2011). *S.* Typhimurium is an excellent model to address antibiotic resistant bacterial persistence and transmittance through the food-chain. The National Antimicrobial Resistance Monitoring System (NARMS) in the United States tracks the information about AR in *Salmonella* including other foodborne pathogens by comparing different bacterial isolates obtained from human infections, animals, and their retail meats. Such information is essential for understanding the effectiveness of antibiotics for both humans and animals (McDermott et al., 2016).

Severe *Salmonella* infections require treatment, and AR is the most serious challenge to treat these bacterial infections (CDC, 2013a, b). The misuse of antibiotics is one of the important factors responsible for high resistance to antibiotics in various pathogenic bacteria, including *S*. Typhimurium (CDC, 2013b). AR in *Salmonella* is linked with horizontal gene transfer and these genes are found on mobile genetic elements. The expansion of antibiotic resistant *Salmonella* serovars are efficient in worldwide dissemination (Butaye et al., 2006; Michael et al., 2006; Alcaine et al., 2007; Mather et al., 2013). *In vitro* antibiotic susceptibility testing is an important basis for monitoring antibiotic susceptibility and resistance trends and for guiding effective anti-infective therapy.

Due to the lack of studies on how the antibiotic resistant *S.* Typhimurium isolates persist and distribute across the food-chain, the objective of this study was to analyze the minimum inhibitory concentrations (MIC) data of different clinically relevant classes of antibiotics of 11,447 *S.* Typhimurium strains from humans, food animals and their products in the United States collected between 1996 and 2016, to investigate the AR pattern through time, food-chain, host origins and also to recommend effective therapeutic options against *S.* Typhimurium infections.

## Materials and Methods

### *Salmonella* Typhimurium Strains

We analyzed available data of *S*. Typhimurium strains obtained from the US NARMS (CDC and NARMS, 2019) database for Enteric Bacteria obtained from 1996 through 2016. Total 11,447 *S*. Typhimurium strains used to analyze in this study were from humans (*n* = 6381), animals (*n* = 2940), and retail meats (*n* = 2126). The *S*. Typhimurium strains from animals were from chickens (*n* = 1396), bovines (*n* = 810), porcine (*n* = 609) and turkeys (*n* = 125). No samplings from ready-to-eat foods and non-animal origin were reported in the current dataset. All the bacterial isolate has clear information of year of isolation, while only around 11% of isolate has clear location information, such as US state of isolation. The aim of this study is to understand the dynamic (time-scale) feature of *S.* Typhimurium across food-chain (animal, retail meat, and human).

The “food-chain” or “food-chain transmission” defined in this study is all about three steps from an infectious disease point of view. The recognized chain for transmission is from animal to animal product, and then to humans, this is how we design the study. We did not study the spread of foodborne pathogens at different stages of industrial food production and processing.

### MIC Determination of Antimicrobial Agents

The minimum inhibitory concentration of the antimicrobial agents tested was recorded for each isolate and compared to breakpoints that were defined by the CLSI when available; otherwise, breakpoint interpretations from the National Antimicrobial Resistance Monitoring System were used as described in the NARMS 2012–2013 annual integrated report (FDA, 2015; Clinical and Laboratory Standards Institute [CLSI], 2017).

The MIC values interpreted according to the CLSI guidelines (Clinical Laboratory Standards Institute [CLSI], 2017) for 27 antibiotics were evaluated and analyzed in this study. These 27 antibiotics were Amikacin (AMI), Apramycin (APR), Gentamicin (GEN), Kanamycin (KAN), Streptomycin (STR), Amoxicillin-clavulanic acid (AMC), Piperacillin-tazobactam (PTZ), Cephalothin (CEP), Cefoxitin (FOX), Ceftriaxone (AXO), Ceftiofur (TIO), Ceftazidime (CAZ), Cefotaxime (CTX), Cefotaxime/clavulanic acid (CTC), Cefquinome (CEQ), Cefepime (FEP), Sulfamethoxazole (SMX), Sulfisoxazole (FIS), Sulfamethoxazole-trimethoprim (COT), Azithromycin (AZM), Aztreonam (ATM), Imipenem (IMI), Ampicillin (AMP), Chloramphenicol (CHL), Ciprofloxacin (CIP), Nalidixic acid (NAL), Tetracycline (TET).

### Statistical Analysis

In this study, we used Random Forest Algorithm and Hierarchical Clustering analysis to classify *Salmonella* Typhimurium population based on their MIC patterns. Recent work demonstrated the usefulness of random forest method because of its unique advantages (Pan et al., 2018). Random Forest package 4.6 was used in this study for the classification. The data set for random forest analysis was classified based on three source (animal, meat, and humans) and five hosts (chicken, bovine, swine, turkey, and human). The input variable was the data set of MIC distribution. The test feature was the MIC values of each individual strains. The strains were categorized into four groups according to the time of their isolation as period 1 (Year 1996–2000), period 2 (Year 2001–2005), period 3 (Year 2006–2010) and period 4 (Year 2011–2016). Based on these four time periods, the diversity of *S.* Typhimurium population was segregated using Random Forest. The MIC values of 27 different antibiotics were used to group different population. Single dot depicts individual strains. The rule for each randomly created decision trees was based on the MIC cut-off values and the vote count was denoted by present (MIC higher than cut off) or absent (MIC lower than cut off). Proximity computation was done on three scales. To identify the best classifier, we have used Classification and Regression Tree (CART) method. Both Random Forest and CART were used to identify the most crucial variables. Using multinomial logistic regression (Afema et al., 2015), here we analyzed antibiotic-susceptibility and antibiotic resistant profiles of *S.* Typhimurium population.

## Results

### Isolates From Different Hosts Behave Differently

**FIGURE 1:**
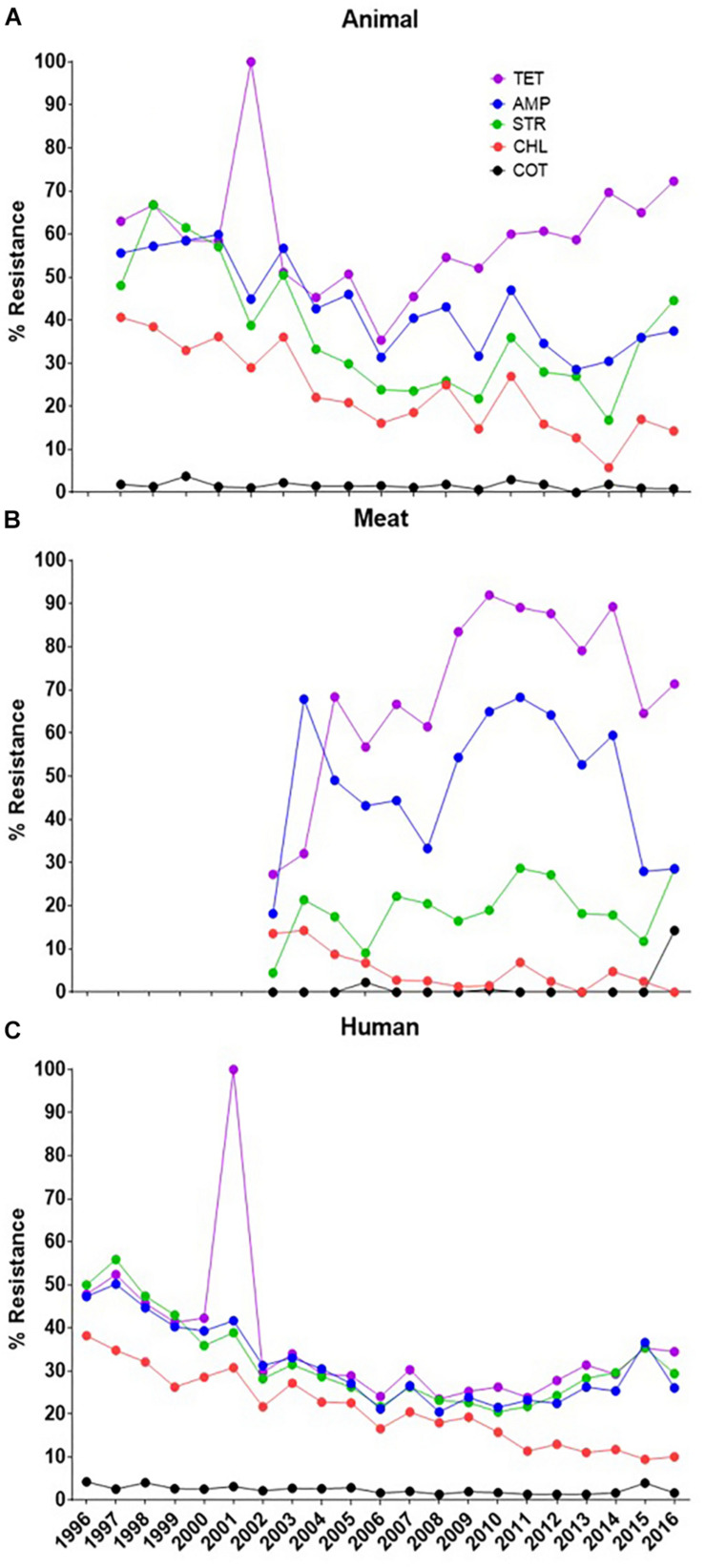
General trend analysis of antibiotic resistance for some certain commonly used molecules (denoted by the color of the lines as given in legend). **(A)** The resistance pattern in animal isolates, **(B)** the resistance pattern in meat isolates and **(C)** the resistance pattern for the human isolates. The XX’ presents the time of data collection while YY’ gives the resistance percent. [Tetracycline (TET), Ampicillin (AMP), Streptomycin (STR), Chloramphenicol (CHL), Sulfamethoxazole-trimethoprim (COT)].

A general trend of the AR of the *S*. Typhimurium isolates from animals, meat and humans to some commonly used antimicrobial agents is presented in Figure 1. In the early 2000s, a surge in resistance to tetracycline by isolates from humans coincided with a similar surge in the animals’ isolates. These results apparently convince that food animals may serve as a pool of antibiotic resistant organisms to humans. But in absence of the records of the simultaneous meat samples a direct link between the animals and humans is missing. For the other years (after 2002) when data is available in all three hosts, the resistance pattern has remained in a generally steady state with a higher rate in animals/meat and a lower rate in humans. In this context, our major focus in this analysis was to show that the isolates from different hosts behave differently.

### Recent Diversification of Antibiotic Resistant Strains

**FIGURE 2:**
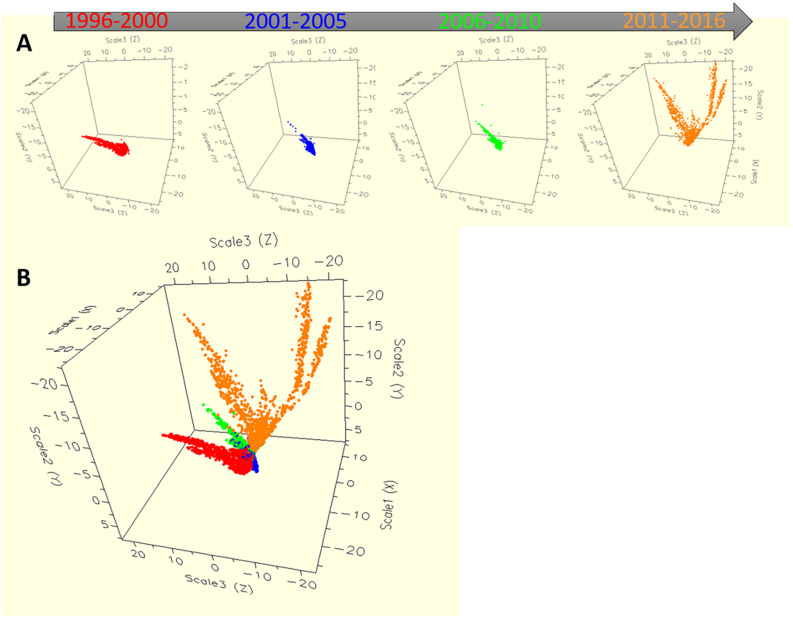
The dynamics of antibiotic resistance features of *Salmonella* Typhimurium strains across 21 years in the United States. The multidimensional scaling plot of 11,447 *Salmonella* Typhimurium strains with MIC values for each bacterial isolate were used for producing the proximity matrices, where *x*-, *y*- and *z*- axes are the multidimensional scaling coordinates. Bacterial isolates with similar MIC values are represented by points close one to the other, whereas isolates with dissimilar antibiotic resistant MIC values are represented by separated points. Each bacterium was indicated as individual dot, where colors represented four different periods. **(A)** The dynamics of antibiotic resistance for four separate time periods. Time period 1 from the year 1996 to 2000, time period 2 from the year 2001 to 2005, time period 3 from the year 2006 to 2010 and time period 4 from the year 2011 to 2016. **(B)** The dynamics of antibiotic resistance for all four time periods describing together.

Based on the four time periods (1996–2000, 2001–2005, 2006–2010, 2011–2016), the diversity of *S.* Typhimurium population was segregated using RandomForest (Figure 2). The MIC values of 27 different antibiotics were used to group different population. Single dot depicts individual strains, with four different colors pointing isolates from four different periods of time. The Figure 2A showed the temporal distribution of the *S*. Typhimurium strains for four time periods from 1996 till 2016. While the distribution based on the MIC of individual strains for until the year 2010 was clustered, the divergence was seen after 2011. For the period 2011–2016, two distinct clusters of the strains are typically visible (Figures 2A,B). It shows that there could have been a shift in the MIC patterns of the isolates after 2011 so they started being divergent.

### Antibiotic Susceptibility and Resistance Analysis

**FIGURE 3:**
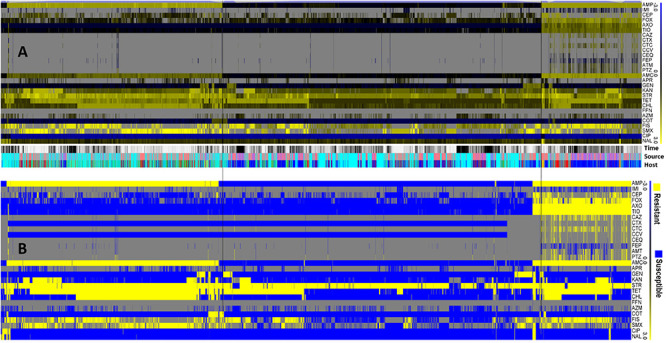
The population diversity of 11,447 *S*. Typhimurium strains from humans, animals, and retail meats. **(A)** Population diversity of *S*. Typhimurium strains grouped by hierarchical clustering. A hierarchical tree with 200 bootstrapping, by using the MIC value of 27 antibiotics, was used to group different population. **(B)** The antibiogram for individual *S*. Typhimurium strains were shown, with blue indicating the susceptibility, and yellow indicating the resistance, based on the MICs interpreted by the CLSI-2017 standards. Gray color indicates strains without MIC value.

Antibiotic resistance analysis with high discriminatory capability allowed differentiation of the 11,447 strains of *Salmonella* Typhimurium. Using hierarchical clustering, population diversity of *S*. Typhimurium was grouped in this study (Figure 3). The log values of the MIC of different antibiotics were used to group Typhimurium population (Figure 3A) which showed the diversification within *S*. Typhimurium strains. The blue to yellow color of the heatmap describing the MICs of individual *S*. Typhimurium strain for every antibiotic. The yellow color pointing the resistance, and blue color pointing the susceptibility. Gray color pointing the strains lacking MIC value (Figure 3B). We found that the resistance was very low against the fluoroquinolones and the resistance was high against ampicillin, chloramphenicol, streptomycin, sulfonamides, tetracycline, amoxicillin-clavulanic acid, ceftriaxone, ceftiofur antibiotics (Figure 3B).

### ASSuT and ACSSuT Are the Most Frequently Found Antibiotic Resistance Patterns

**FIGURE 4:**
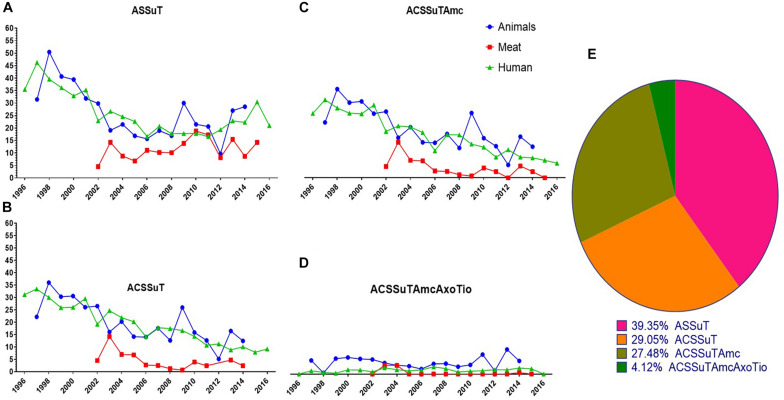
Graphical representations of four antibiotic resistance patterns (ASSuT, ACSSuT, ACSSuTAmc and ACSSuTAmcAxoTio) found in *S*. Typhimurium strains through 1996–2016 in United States. **(A)** ASSuT (Ampicillin, Streptomycin, Sulfonamides, and Tetracycline) resistance patterns found in *S*. Typhimurium isolates from animals, meat and humans. Though the ASSuT resistance in animal and human isolates of Typhimurium declined sharply during 2002–2008, the resistance is on rise in the recent years. The ASSuT resistance in meat isolates showed increasing pattern with time. **(B)** ACSSuT (Ampicillin, Chloramphenicol, Streptomycin, Sulfonamides, and Tetracycline) resistance patterns found in *S*. Typhimurium isolates from animals, meat and humans. The ACSSuT resistance in human and meat isolates of Typhimurium showing decreasing pattern but the same resistance in animal isolates showing increasing trend with the time. **(C)** ACSSuTAmc (Ampicillin, Chloramphenicol, Streptomycin, Sulfonamides, Tetracycline, and Amoxicillin-clavulanic acid) resistance patterns found in *S*. Typhimurium isolates from animals, meat and humans. The ACSSuTAmc resistance in human and meat isolates of Typhimurium showing decreasing pattern but in animal isolates the resistance remained high with time. **(D)** ACSSuTAmcAxoTio (Ampicillin, Chloramphenicol, Streptomycin, Sulfonamides, Tetracycline, Amoxicillin-clavulanic acid, Ceftriaxone, and Ceftiofur) resistance patterns found in *S*. Typhimurium isolates from animals, meat and humans. ACSSuTAmcAxoTio resistance in animal isolates showed increasing tendency in the recent years. The XX’ presents the time of data collection while YY’ gives the percent of resistance. **(E)** Pie chart showing the percentage of distribution of four different resistance patterns among 7237 isolates of *S*. Typhimurium from animals, meat and humans.

**FIGURE 5:**
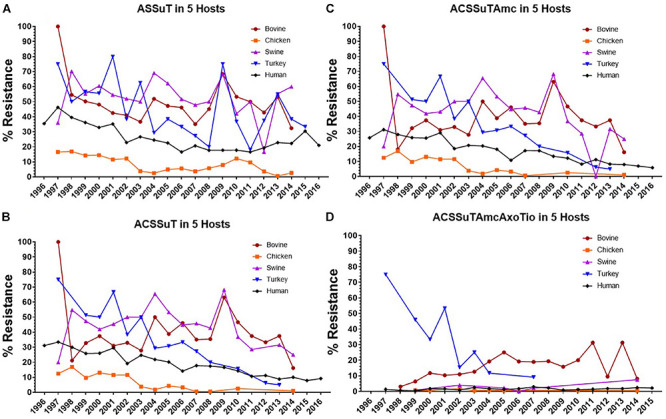
Graphical representations of four antibiotic resistance patterns (ASSuT, ACSSuT, ACSSuTAmc and ACSSuTAmcAxoTio) found in *S*. Typhimurium isolates through 1996–2016 in United States obtained from five different hosts (bovine, chicken, swine, turkey, and human). **(A)** ASSuT (Ampicillin, Streptomycin, Sulfonamides, and Tetracycline) resistance patterns found in *S*. Typhimurium isolates from bovine, chicken, swine, turkey and human. *S*. Typhimurium isolates from bovine, swine and turkey showed higher percentage of this resistance pattern than the isolates from chicken and human. **(B)** ACSSuT (Ampicillin, Chloramphenicol, Streptomycin, Sulfonamides, and Tetracycline) resistance patterns found in *S*. Typhimurium isolates from bovine, chicken, swine, turkey and human. Swine and bovine isolates showed more ACSSuT resistance pattern than other host isolates. **(C)** ACSSuTAmc (Ampicillin, Chloramphenicol, Streptomycin, Sulfonamides, Tetracycline, and Amoxicillin-clavulanic acid) resistance patterns found in *S*. Typhimurium isolates from bovine, chicken, swine, turkey and human. Swine and bovine isolates showed more percentage of resistance pattern among all host isolates. **(D)** ACSSuTAmcAxoTio (Ampicillin, Chloramphenicol, Streptomycin, Sulfonamides, Tetracycline, Amoxicillin-clavulanic acid, Ceftriaxone, and Ceftiofur) resistance patterns found in *S*. Typhimurium isolates from bovine, chicken, swine, turkey and human. Percentage of this resistance pattern found high in bovine Typhimurium isolates. The XX’ presents the time of data collection while YY’ gives the percent of resistance.

The AR patterns found in human isolates were compared with the AR patterns of isolates from animals and retail meats. Interestingly, the most frequently observed resistance patterns were tetra-resistant pattern ASSuT (Ampicillin, Streptomycin, Sulfonamides, and Tetracycline) and penta-resistant pattern ACSSuT (Ampicillin, Chloramphenicol, Streptomycin, Sulfonamides, and Tetracycline). Other AR patterns such as ACSSuTAmc (Ampicillin, Chloramphenicol, Streptomycin, Sulfonamides, Tetracycline, and Amoxicillin-clavulanic acid) and ACSSuTAmcAxoTio (Ampicillin, Chloramphenicol, Streptomycin, Sulfonamides, Tetracycline, Amoxicillin-clavulanic acid, Ceftriaxone, and Ceftiofur) were also analyzed in this study. These four patterns comprised of 63% of the total 11,447 isolates. Among these, 39% of isolates showed ASSuT resistance pattern, 29% of isolates showed ACSSuT resistance pattern, 28% of isolates showed ACSSuTAmc and 4% of isolates showed ACSSuTAmcAxoTio resistance pattern. Four AR patterns found in *S*. Typhimurium isolates in this study are shown in Figure 4. Figure 5 showed the graphical representations of antibiotic resistance patterns found in *S*. Typhimurium isolates obtained from five different hosts (chicken, bovine, swine, turkey, and human).

We found that, the ASSuT resistance in animal and human isolates of Typhimurium declined sharply during 2002–2008, but the resistance is on rise in the recent years. The ASSuT resistance in meat isolates showed increasing pattern with time (Figure 4A). *S*. Typhimurium isolates from bovine, swine, and turkey showed higher percentage of this resistance pattern than the isolates from chicken and human (Figure 5A). In this study, high resistance was recorded in *S*. Typhimurium strains to ampicillin, chloramphenicol, streptomycin, sulfonamides, and tetracycline (ACSSuT). The ACSSuT resistance in human and meat isolates of Typhimurium showed a decreasing but the same resistance in animal isolates showed an increasing trend with time (Figure 4B). Swine and bovine isolates showed more ACSSuT resistance pattern than other host isolates (Figure 5B). The ACSSuT pattern also found in different combinations with AMC, AXO, TIO antibiotics as additional resistances in our study. For ACSSuTAmc resistance, the isolates from human and meat showed decreasing pattern, but in animal isolates the resistance remained high with time (Figure 4C). The frequency of ACSSuTAmcAxoTio pattern remained constant over the years, though the resistance in isolates from animal showed a slight increase in the recent years (Figure 4D). ACSSuTAmc pattern was more prevalent in swine and bovine isolates and ACSSuTAmcAxoTio pattern in the bovine isolates (Figures 5C,D). Figure 4E showed the percentage of distribution of four different resistance patterns among 7237 strains of *S*. Typhimurium obtained from animals, meat and humans.

### Analysis of the Best Classifier for Antibiotic Resistance Divergence

Classification and regression tree analysis of individual strains based on the MIC for multiple antimicrobials reveal that the MIC value toward COT (Trimethoprim-sulfamethoxazole) could be the best classifier to identify the origin of the isolates. The isolates originating in the animals and meat had a COT MIC of >0.12250 whereas those originating in the humans were having COT ≤ 0.12250. This implies that in spite of being susceptible to COT, the effective MIC is higher for the animal or meat isolate as compared to the human isolate. Similarly, if the isolates are to be classified based on their host (as humans, chickens, bovine, porcine, and turkey), the chicken isolates could be best identified as those isolates with MIC for COT > 0.12250 (biological cut off is 2), GEN ≤ 6 (biological cut off is 4), and STR ≤ 48 (biological cut off is 16). That means strains susceptible to COT but resistant to gentamicin and streptomycin are likely to have originated from chickens.

### Sources for Human Infections and Dissemination of Resistance Genes

**FIGURE 6:**
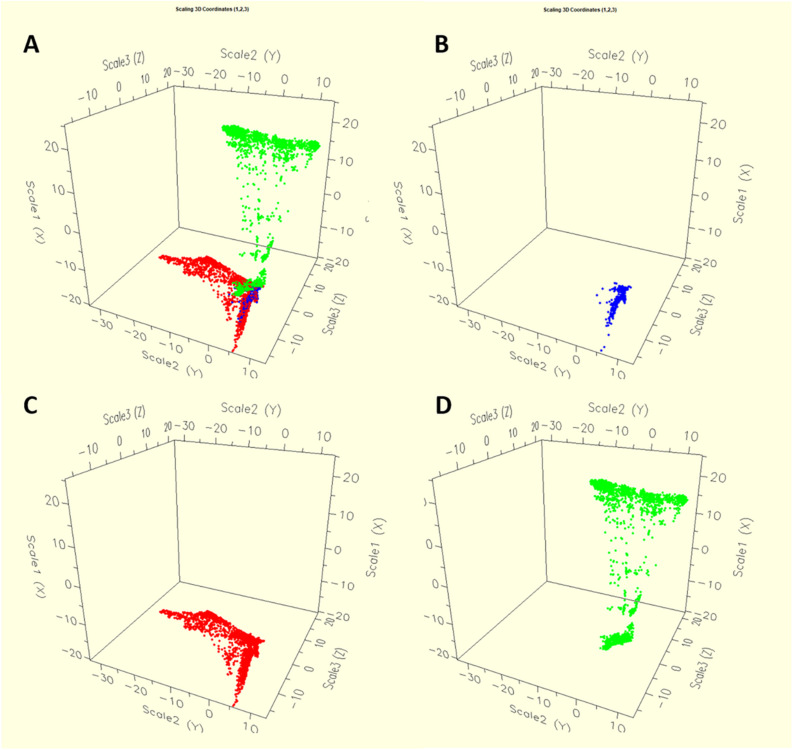
The dynamics of antibiotic resistance features of *Salmonella* Typhimurium isolates according to the all sources **(A)** such as animal **(C)**, meat **(B)** and human **(D)** used in this study. The diversity of antimicrobial resistance is greater in the isolates from the human population than in those from animals and animal meat.

**FIGURE 7:**
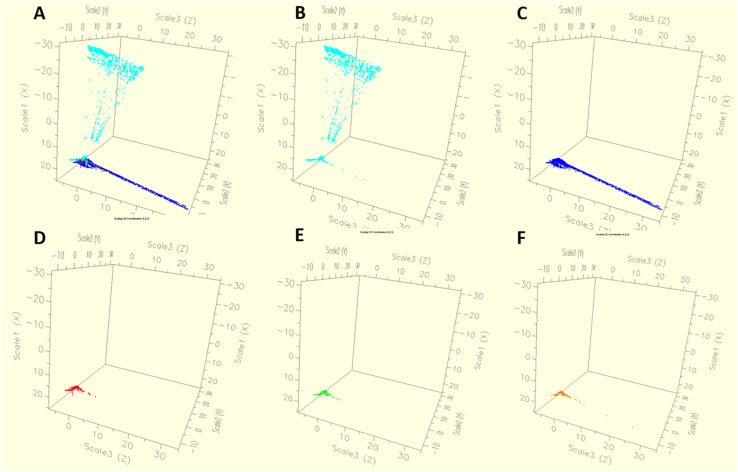
The dynamics of antibiotic resistance features of *Salmonella* Typhimurium isolates by host population **(A)** such as Human **(B)**, Chicken **(C)**, Bovine **(D)**, Porcine **(E)**, Turkey **(F)**. *S*. Typhimurium strains from human and chicken showed high antibiotic resistance diversity more than bovine, porcine and turkey strains.

We found considerable variation in the resistance profile of *Salmonella* Typhimurium obtained from different sources. The AR profile abundance was higher in *S*. Typhimurium isolates obtained from humans. We found that *S*. Typhimurium strains from human and animal were emerged in easily distinguishable populations. Antibiogram of the strains derived from animals showed less diverse than the strains derived from humans. This significantly suggests that multiple sources could be involved in *Salmonella* infections in human. Our result also described that, epidemics of the human and animal are not similar and the diversity of AR is greater in the human isolates than the diversity of AR in isolates derived from animals and animal meat (Figure 6). We also found that *S*. Typhimurium strains from chicken showed higher AR diversity than the bovine, porcine or turkey strains (Figure 7).

## Discussion

Continuous monitoring of the emergence of any bacterial serotype to detect in the food-chain is very important for public health point of view globally. The *Salmonella* Typhimurium strains from the recent years (2011–2016) showed much AR diversification as compared to previous year ranges (Figure 2). This is interesting and it could be explained by the fact that, the use as well as misuse of antibiotics is on rise in recent years and as a result antibiotic resistant strains of *S*. Typhimurium increased sharply and transmission of these strains occurred in the food-chain which ultimately affects humans. The immense use of antibiotics could be the main reason behind the emergence of AR in *S*. Typhimurium strains.

The reduced susceptibility pattern showed by *S*. Typhimurium population (Figure 3) could be responsible for treatment failures in some clinical situations. Streptomycin is not regularly used for the treatment of salmonellosis; but it is commonly used as a growth promoter in animals. Due to this reason, streptomycin could serve as a marker for resistant isolates moving through the food-chain (McDermott et al., 2016). Among the multiple factors that confer the emergence of antibiotic resistant bacteria, the extensive and overuse of antibiotics in medical system and agriculture is believed as the most pivotal (NARMS, 2019). The mechanism of the AR in *Salmonella* at the cellular level is complex (Penesyan et al., 2015). *Salmonella* develops AR mechanisms by the production of enzymes that can destroy action of antibiotics, by activating efflux pumps, and by producing β-lactamase which can degrade the structure of antibiotic molecules (Foley and Lynne, 2008; Andino and Hanning, 2015). AR can also be achieved by biofilm production which can cause increased risk of food safety (Steenackers et al., 2012; Corona and Martinez, 2013).

A very important feature of DT104 is that it is bearing *Salmonella* genomic island 1 (SGI-1). SGI-1 contains different AR genes identified in several *Salmonella enterica* serovars (Mossoro-Kpinde et al., 2015). Mulvey et al. (2006) reported that, variation in SGI-1 of the DT104 strains occurs through recombination. Another study using 359 strains demonstrated frequent and recurrent loss or gain of AR genes from the entire SGI-1 to single anti-microbial resistance genes (Mather et al., 2013). One of the characteristics of DT104 is that the strain typically shows resistance to ampicillin, chloramphenicol, streptomycin, sulfonamide, and tetracycline and it has the capability to gain resistance to other clinically important antibiotics (Helms et al., 2005; Mulvey et al., 2006). During 1990s MDR DT104 rapidly emerged globally and became the most common foodborne pathogen found in humans and animals (Ridley and Threlfall, 1998; Threlfall, 2000; Helms et al., 2005). Integrons, which contain AR genes, acquisition is also a very effective mechanism for DT104 to acquire resistance to different antibiotic classes (Stokes and Hall, 1989; Collis and Hall, 1995; Levesque et al., 1995).

Our analysis reveals that resistance was very low to an important group of antibiotics, the fluoroquinolones (Figure 3B). Due to its broad-spectrum activity, ciprofloxacin, which is a fluoroquinolone compound, is used for the treatment of *Salmonella* infections (Fàbrega and Vila, 2013). This same antibiotic is commonly recommended in sub-Saharan Africa, and prescribed with other important fluoroquinolone compounds such as levofloxacin, ofloxacin, and norfloxacin for patients with enteric diseases (Kagambèga et al., 2018). The antibiotic treatment regimen recommended by clinicians against *Salmonella* infection in human includes third generation cephalosporins, quinolones and macrolides (Guerrant et al., 2001). However, as a result of the high frequency of AR of animal-origin *Salmonella* isolates against cephalosporins, the recommended usage of cephalosporins became a great concern for the treatment of *Salmonella* derived from animals which can infect humans as well as other healthy animals. Another two antibiotics such as tetracycline and streptomycin showed high level of AR in this study, suggesting that clinicians should consider avoiding these two antibiotics for clinical use as well as in animal husbandry. The AR analysis in this study suggested fluoroquinolones (i.e., Ciprofloxacin) still can be used as a therapeutic agent of choice against *Salmonella* Typhimurium infection.

Four important AR patterns (ASSuT, ACSSuT, ACSSuTAmc, and ACSSuTAmcAxoTio) were analyzed in *S*. Typhimurium strains in this study (Figures 4, 5). ACSSuT pattern generally used when bacteria become resistance to ampicillin, chloramphenicol, streptomycin, sulfonamides, and tetracycline. A resembling resistance pattern ASSuT is used to show resistance to four antibiotics. Other important phenotypes (ACSSuTAmc and ACSSuTAmcAxoTio) which we found to be more prevalent along with ACSSuT and ASSuT were also analyzed here. The ASSuT resistance pattern was found related with *S*. Typhimurium DT193 strains which caused human infection in countries such as Spain, England and Wales (Prats et al., 2000; Threlfall et al., 2000). This same AR pattern was found associated with porcine population in United Kingdom (Maguire et al., 1993; Hampton et al., 1995). The ASSuT became more usual in Italy in *S*. Typhimurium since 2000 (Graziani et al., 2008; Dionisi et al., 2009). Here, this ASSuT is linked with *S*. Typhimurium strains of animals as well as strains of human origin. Busani et al. (2004) demonstrated a lower frequency of resistance to ACSSuT pattern in *S*. Typhimurium isolates obtained from humans than from non-human isolates. The ACSSuT is the typical resistance pattern factors of the DT104 (Adhikari et al., 2010; Mateva et al., 2018). The MDR *S*. Typhimurium strains in other studies showed similar AR pattern (ACSSuT); (Ribot et al., 2002; De Vito et al., 2015; Mossoro-Kpinde et al., 2015). Some reports described that the AR type ACSSuT may have evolved in Asia around early 1980s in other pathogens and were then horizontally transferred to DT104 (Angulo et al., 2000; Ribot et al., 2002).

*Salmonella* Typhimurium strains obtained from animal, meat and human were found to originate in distinct clusters. In our study, trimethoprim-sulfamethoxazole was found as the best classifier for AR differentiation of different population, which is also evidenced in other serovar such as *S*. Newport (Pan et al., 2018).

Based on antimicrobial susceptibility testing, our result suggests that multiple sources could be involved in *Salmonella* infections in human (Figure 6). Certain clusters of isolates act as the bridge between these human and animal isolates. From this perspective, the isolates from meat samples are at the interface of both animal and human (Figure 6). A study by Sun et al. (2014) demonstrated that *S*. Typhimurium infection in humans is linked with different clades than those which are prevalent in pet animals. Contaminated meat, milk and eggs are the probable source of *Salmonella* infection in humans. Humans are having

different diverse source of infections as compared to the local animals and it is possible that the AR diversity is more in humans than in animals (Figure 6). In their study, Mather et al. (2012) used epidemiological as well as ecological methods to analyze phenotypic AR data in DT104. The study found greater AR diversity in isolates derived from humans as compared to isolates obtained from animals. In another study, Wiesner et al. (2009), using MLST and PFGE methods found the existence of highly distributed *S*. Typhimurium strains that were derived in Mexico from human and food-animals, and from diverse geographic locations during different years.

The occurrence of antibiotic resistant strains in humans and chicken (Figure 7) warns of the possible risk of consumption of contaminated food. Through contamination of food, poultry may act as a very effective vector for transmission of *Salmonella* to human (Bemrah et al., 2003; Vandeplas et al., 2010; Paine et al., 2014; Li et al., 2018). Periodic outbreaks of live poultry-associated salmonellosis (LPAS) pointed the need of new alternative potential strategies to control human illnesses. LPAS outbreaks in the United States have been documented since 1955 (Anderson et al., 1955) and report showed that, the number of LPAS incidents is on rise significantly in last few years. Most of the LPAS outbreaks start in February (Basler et al., 2016). The DT104 strain which was correlated with cattle gradually became prevalent in poultry, pigs, and wild animals and showed a typical penta-resistance pattern of ACSSuT (Glynn et al., 1998). Study by Zhu et al. (2017) suggested the role of broiler chicken as the reservoir for multi-drug resistant *Salmonella* and in the processing plants cross-contamination is frequent between chicken and the environment including the workers working in the plants. This cross-contamination is of importance and these leads to the enhance of MDR *Salmonella* strains in the final stages which ultimately facilitate the spread of the AR genes widely to the consumers. Bovine salmonellosis is a significant threat to beef industry (Wray and Davies, 2000). Pigs are also responsible for the transmission of *Salmonella* to humans (Barilli et al., 2018). As the *S.* Typhimurium sometimes asymptomatic in pigs, most of the time the pathogen does not cause illness in pigs, but pigs could be an important source of contamination of the dead animals in the slaughter houses (Davies and Wray, 1997). This bacterial contamination could not be detected in the pigs in the farm, but this could finally cause the transmission to human (Kich et al., 2011; Almeida et al., 2016). Very few reports detected the antibiotic resistant *Salmonella* in turkey meat (Fakhr et al., 2006; Khaitsa et al., 2007) which is supporting our findings.

## Conclusion

Our results revealed some significant observations for *Salmonella* Typhimurium epidemiology describing AR pattern dynamics across the food-chain. The recent diversification of AR found among the *S*. Typhimurium isolates is of great importance and significantly draw attention about the misuse of the antibiotics in the recent years. Major resistance patterns found were tetra-resistance and penta-resistance. The population diversity analysis calculated based on the MIC value revealed typical patterns of host segregation suggesting the distinction between the isolates from different host and sources but with some cross-overs and common shared population. This study helped in understanding the expansion of dynamics of antibiotic resistance of *S*. Typhimurium and recommended fluoroquinolone antibiotics as a possible potential therapeutic option against *S*. Typhimurium infection. The antimicrobial susceptibility, resistance profiles and different AR patterns in *Salmonella* strains including other potential pathogenic and virulence factors is expanding globally and this phenomenon must be monitored cautiously and continuously for the betterment of the treatment of salmonellosis. Nevertheless, this study has some limitations. The sampling bias is the one, with over sampling in human and less in animals, including disequilibrium in samples of animal origins. Most of the data presenting in this study is based on the quantitative MICs data. Future study based on genetic information, particularly whole genome sequence, can accelerate our understanding of *S.* Typhimurium population diversity and transmission along the food-chain.

## Author Contributions

XW and SB contributed equally to this work, conducted the data analysis, and wrote the manuscript. HP and NP made significant contribution to refine and reorganized the data used in the manuscript and its presentation. XL and WF provided critical comments and aided with the edition of the manuscript. MY conceived the idea, collected the data, and suggested the statistical interpretation of the data analyzed and aided with the writing. All authors have read and approved the manuscript.

## Conflict of Interest Statement

The authors declare that the research was conducted in the absence of any commercial or financial relationships that could be construed as a potential conflict of interest.
