# Corrigendum: Antibiotic Resistance in *Salmonella* Typhimurium Isolates Recovered From the Food Chain Through National Antimicrobial Resistance Monitoring System Between 1996 and 2016

**DOI:** 10.3389/fmicb.2020.01738

**Published:** 2020-08-14

**Authors:** Xuchu Wang, Silpak Biswas, Narayan Paudyal, Hang Pan, Xiaoliang Li, Weihuan Fang, Min Yue

**Affiliations:** ^1^Hangzhou Center for Disease Control and Prevention, Hangzhou, China; ^2^CATG Microbiology and Food Safety Laboratory, Institute of Preventive Veterinary Medicine, College of Animal Sciences, Zhejiang University, Hangzhou, China; ^3^Zhejiang Provincial Key Laboratory of Preventive Veterinary Medicine, Hangzhou, China

**Keywords:** *Salmonella* Typhimurium, foodborne pathogen, antibiotic resistance, minimum inhibitory concentration, population diversity, fluoroquinolones

In the original article, the Acknowledgments section was not included. This section appears below.

